# An Unusual Case of Flank Pain Late in Life: A Case Report

**DOI:** 10.7759/cureus.52790

**Published:** 2024-01-23

**Authors:** Kristel Sibaja, Harper Henderson, Alejandro Biglione, RaeAnn Tourangeau-Young

**Affiliations:** 1 Dr. Kiran C. Patel College of Osteopathic Medicine, Nova Southeastern University, Davie, USA; 2 Internal Medicine, Wellington Regional Medical Center, Wellington, USA

**Keywords:** acute pain, kidney anomaly, fused kidney, congenital anomalies of the kidney and urinary tract (cakut), flank pain, horse shoe kidney

## Abstract

Abnormalities in renal fusion represent a subset of congenital anomalies of the kidneys and urinary tract (CAKUT). Horseshoe kidneys (HSKs) are the fusion of kidneys at their lower poles. It is the most common form of CAKUT. Symptoms are usually subtle. The diagnosis is usually made incidentally during childhood. Rarely does an HSK become symptomatic later in life. We present the case of an 88-year-old female with a history of HSK who presented to the emergency department (ED) with a three-week history of left-sided flank pain, intermittent nausea, and reduced urine output. Her inpatient workup included imaging that revealed an HSK and bilateral hydronephrosis, which was more prominent on the left. The onset of symptoms for an HSK late in life is extremely rare.

## Introduction

The most common subset of congenital anomalies of the kidneys and urinary tract (CAKUT) are abnormalities of the fusion process. Horseshoe kidney (HSK) is the most common form caused by interference in the embryological pathway of the formation of the kidneys. It results in a connection at the inferior poles of the kidneys due to the amalgamation of the mesenchyme or Wolffian ducts [1]. HSK occurs in approximately 0.25% of the general population. However, its frequency is heavily influenced by the test used for the diagnosis, therefore making it difficult to determine a true incidence. It occurs more frequently in males in a 2:1 ratio. It is found in more than 50% of patients with Edward syndrome, up to 20% with Turner's syndrome, and 1% with Down syndrome. It is frequently asymptomatic and is most common in children who receive imaging for other medical conditions or the workup for urinary complaints [2,3]. It is rare for an HSK to become symptomatic with the first complication late in life.

## Case presentation

We present the case of an 88-year-old female who presented to the emergency department (ED) with a three-week history of left-sided flank pain. The pain was dull, of severe intensity, non-radiating, intermittent, and associated with nausea and heaving. She denied fever, chills, shortness of breath, chest pain, dysuria, and frequent urination.

She had an extensive past surgical history, including four c-sections, hysterectomy, cholecystectomy, right bunionectomy, left-hand ganglion cyst removal, and two left myringomethomies with tympanostomy tubes. In terms of social history, she was a non-smoker, consumed wine once or twice a month, and denied substance abuse. She lived independently in her own home and consumed a well-balanced diet.

Upon questioning her family history, it was revealed that her father had an acute myocardial infarction, and her mother had lung cancer. In addition, her brother had a history of a stroke, and her sister had type II diabetes mellitus.

**Figure 1:**
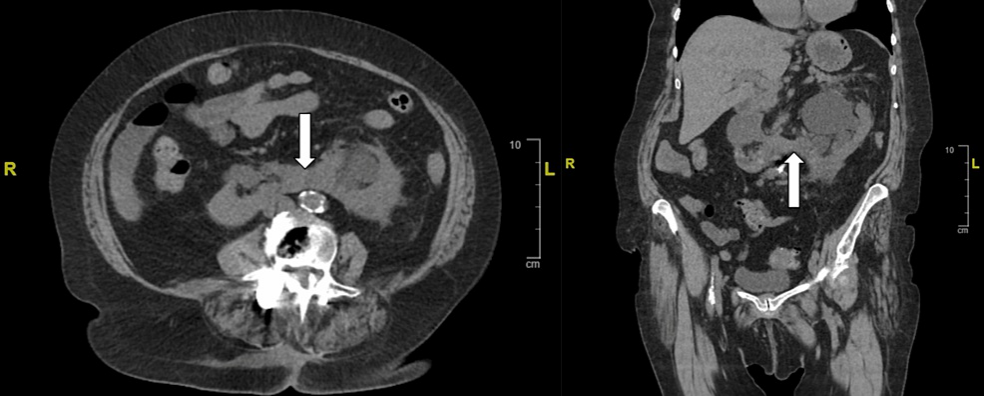
CT abdomen/pelvis without contrast in axial (left) and coronal (right) views showed an HSK (white arrows). HSK: Horseshoe kidney

**Figure 2:**
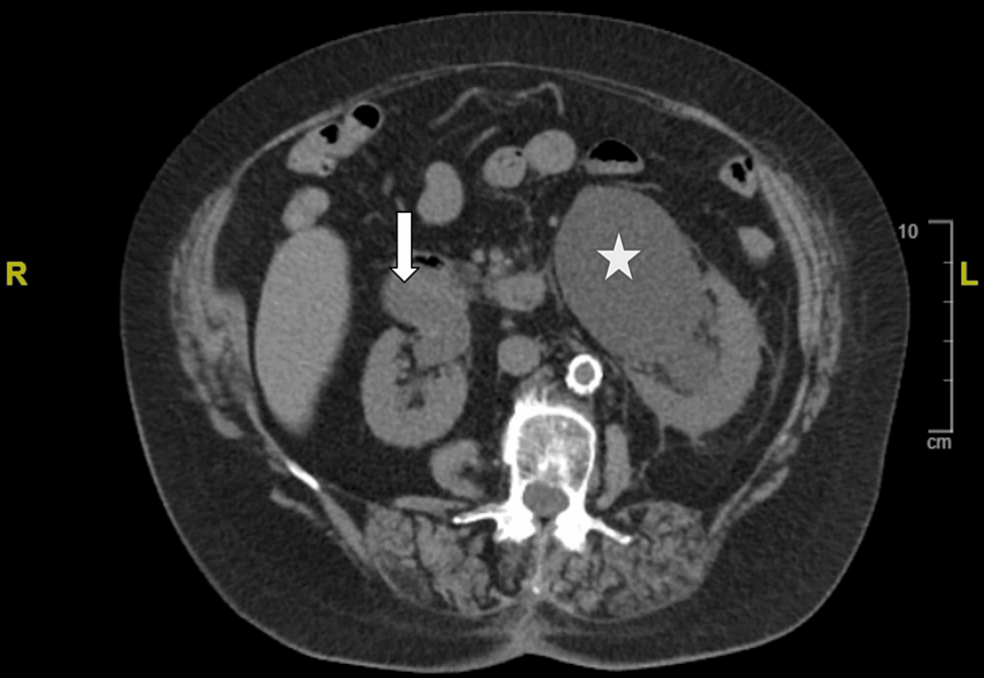
CT abdomen/pelvis without contrast in axial view shows bilateral hydronephrosis more prominent on the left (white star) compared to the right (white arrow).

**Figure 3:**
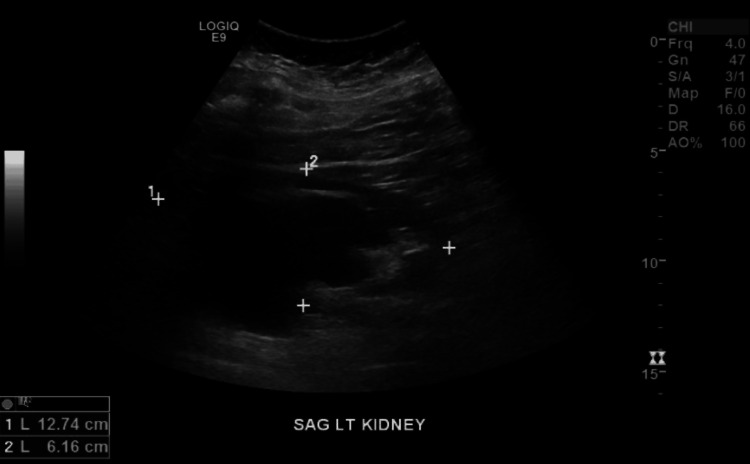
Complete renal ultrasound showed bilateral hydronephrosis, prominently worse on the left.

**Figure 4:**
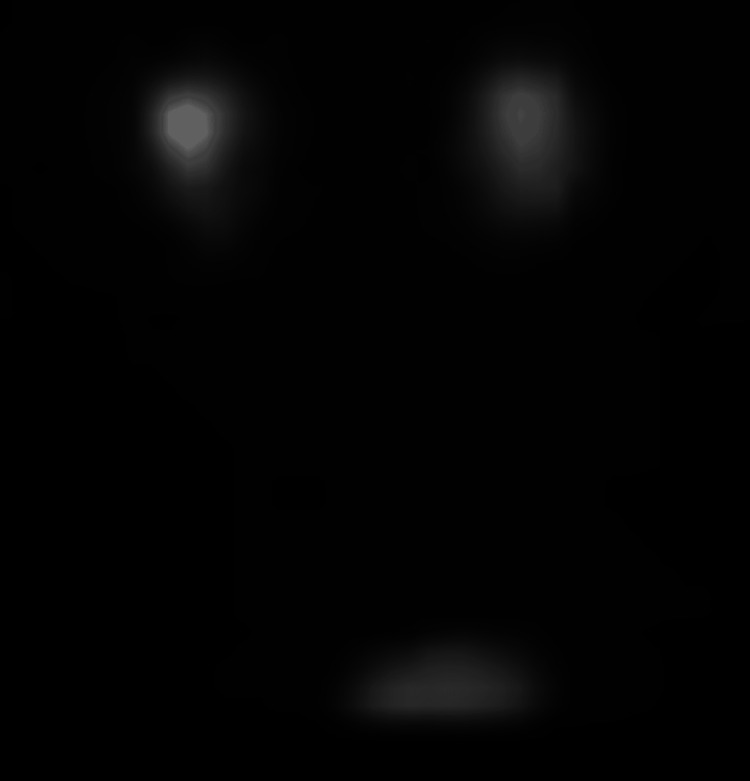
Nuclear medicine renal scan with flow shows intact fluid passage from kidney to bladder bilaterally.

**Figure 5:**
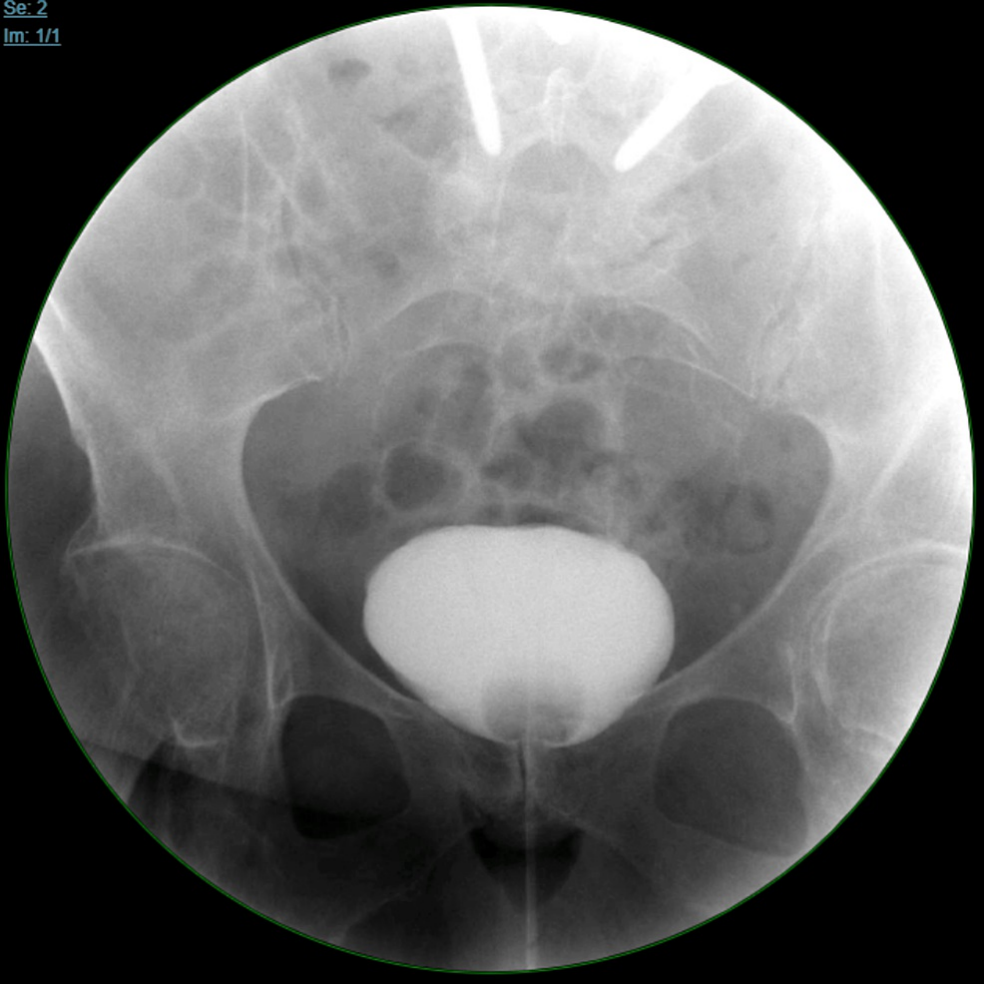
X-ray voiding urethrocystography (VCUG) revealed no reflux into the ureters.

A CT of the abdomen and pelvis without contrast confirmed the presence of an HSK (Figure 1) and bilateral hydronephrosis, more prominent on the left (Figure 2). Ultrasound of the kidneys revealed bilateral hydronephrosis with a left predominance (Figure 3). A nuclear medicine renal flow scan with furosemide was ordered to visualize the function of the whole HSK. The scan showed bilateral excretion with asymmetric renal split in function, 60% on the right and 40% on the left (Figure 4). A voiding urethrocystography showed no bladder abnormalities and no evidence of vesicoureteral reflux (VUR) as shown in Figure 5.

A cause for the hydronephrosis could not be found. Therefore, through shared decision-making, the urologist recommended conservative medical management with as-needed analgesics to ease pain, and the patient opted for no surgical interventions. The patient was discharged on oral analgesics with instructions to follow up closely with a urology outpatient for ureteral stenting if the pain persists.

## Discussion

Most patients with an HSK are asymptomatic and diagnosed incidentally through imaging. Symptomatic patients usually present in childhood with abdominal pain, with or without hematuria, secondary to a urinary obstruction or infection [1]. Early detection requires a high level of suspicion and serves to forestall potential complications. Only in rare cases do patients develop symptoms later in life as their first occurrence of a complication. The most common symptom is pain related to obstruction. The most common site of obstruction is distal, and it is because of congenital ureteropelvic junction obstruction (UPJO). A probable cause for UPJO is the elevated positioning of the ureters as they enter the renal pelvis and the repositioning of the fused isthmus [4]. This orientation puts patients with an HSK at increased risk for obstruction and infections. 

This orientation and change in anatomy can lead to atypical presentations of common diseases. Pyelonephritis typically presents with flank pain and/or costovertebral angle tenderness on physical examination. Yokose et al. presented a case of an 83-year-old patient with abdominal pain who was sent home from the ED after a workup was negative. This led to a delayed diagnosis when a positive blood culture returned and a CT showed an HSK with right hydronephrosis and fat stranding [5]. This change in anatomy also puts patients at risk of trauma since the HSK sits lower in the abdomen and is not protected by the ribs. In addition, there is an increased risk of fracture in the lumbar vertebrae where the isthmus crosses the midline [3].

The evaluation of a patient with an HSK includes a thorough physical exam to evaluate for urogenital abnormalities. Evaluation of renal function is an integral part of the workup. A urinalysis should be obtained to evaluate for proteinuria and hematuria. Renal imaging should be obtained and can include ultrasound, CT, and/or MRI scans of the abdomen and pelvis. They reveal the abnormal morphology of the kidneys, such as complications like hydronephrosis and associated urogenital malformations. These imaging tests may also aid in finding any contributing factors to symptoms, such as any stones or other causes of hydronephrosis. In a 2018 meta-analysis, they found kidney stones to have an incidence of 36% in these patients' lifetimes [6]. Another source of clinical manifestations may be VUR, which can contribute to an increased incidence of infection. In a case series of 52 individuals with an HSK, VUR was found in 25% of patients, UPJO in 23% of patients, and an ectopic ureter in one patient [7].

HSK can be a feature of many syndromes and related structural anomalies. In a retrospective study of 380 patients with an HSK, 50% (n=190) of patients were found to have extrarenal diseases or syndromes, with the most common being gastrointestinal tract anomalies in 16.3% (n=62) and vertebral anomalies in 14.2% (n=54). In addition, 49 patients (12.9%) were found to have syndromes, with Turner's syndrome being found in 16 patients (4.2%) [4]. Nuclear medicine scans can evaluate renal function, particularly in cases of hydronephrosis when urologic repair is anticipated. Voiding cystourethrography is useful for certifying VUR.

In our patient, we were not able to find a cause for her hydronephrosis; therefore, we opted for conservative management. If found, however, then treat the underlying problem. UPJO can be treated with either percutaneous nephrostomy (PCN), ureter stenting, or surgically with open urethroplasty or laparoscopic dismembered pyeloplasty. Nephrolithiasis treatment options are extracorporeal shockwave lithotripsy and open surgery or PCN as a temporizing measure or palliatively for symptom relief. If due to infection, treat with antibiotics while also ruling out risk factors such as stasis, reflux, and stone formation [8]. In patients who have repeated, chronic complications with no relief, heminephrectomy can sometimes be the answer [9].

Yohannes et al. presented two cases that ultimately led to a heminephrectomy. The first case was of a 56-year-old due to a urinary tract infection and a nonfunctioning, left hydronephrotic kidney with 0% flow seen on a nuclear medicine scan. The second case was of a 48-year-old who had a hydronephrotic left kidney on ultrasound, markedly atrophic with fat stranding on the CT scan, and only 2% functioning on the nuclear medicine scan [10]. In our patient, however, both of her kidneys were still functioning with 60% on the right and 40% on the left. This is also a more aggressive option that an elderly patient may not want to do.

## Conclusions

The onset of symptoms from an HSK in the ninth decade of life, as witnessed in our patient, is a rare occurrence. An extensive test battery and comprehensive imaging studies were meticulously conducted to ascertain the underlying cause of her initial complaint of flank pain. A heightened level of awareness should be maintained to prevent complications.
